# SOCIAL JET LAG: SOCIODEMOGRAPHIC DETERMINANTS AND HEALTH IMPLICATIONS IN MIDDLE-AGED AND OLDER ADULTS

**DOI:** 10.1093/geroni/igae098.1179

**Published:** 2024-12-31

**Authors:** Hanamori Skoblow, Eunjin Tracy, Eunjung Kim, Naomi Meinertz, Getrude Nyang’au, Megan Gilligan

**Affiliations:** University of Missouri, Columbia, Missouri, United States; University of Missouri, Columbia, Missouri, United States; Arizona State University, Tempe, Arizona, United States; University of Missouri, Columbia, Missouri, United States; University of Missouri, Columbia, Missouri, United States; University of Missouri, Columbia, Missouri, United States

## Abstract

Social jet lag is characterized by inconsistent sleep patterns between weekdays and weekends and arises from a misalignment between biological rhythms and social schedules, posing significant health risks. However, the sociodemographic determinants and health implications of social jet lag among middle-aged and older adults are unclear. In the current study, we examined cross-sectional associations between social jet lag, sociodemographic characteristics, and key mental, physical, and cognitive health outcomes. Among adults ages 50–83 (M = 68.22; SD = 7.85) in the 2018 wave of the Health and Retirement Study (N = 667; 62% women), approximately 49% of respondents experienced social jet lag. Bivariate correlations indicated that social jet lag was related to older age (r = –.35, p <.001), Hispanic ethnicity (r =.13, p =.001), and current employment (r =.31, p <.001), but not gender (p =.99) or education (p =.12). Results of multivariate regressions, controlling for the above sociodemographic determinants, indicated that greater social jet lag was associated with higher depressive symptoms (β = 0.14, p <.001, R-squared =.10) and lower cognitive performance (β = –0.08, p =.04, R-squared =.24). Social jet lag was not significantly associated with body mass index (p =.36), number of chronic conditions (p =.59), or self-rated memory (p =.72). These findings point to the importance of a regular sleep schedule as a modifiable behavior that may promote better mental and cognitive health in midlife and beyond.

